# Antimicrobial Resistance and Biofilm Formation Capacity of *Salmonella enterica* Serovar Enteritidis Strains Isolated from Poultry and Humans in Poland

**DOI:** 10.3390/pathogens9080643

**Published:** 2020-08-07

**Authors:** Katarzyna Ćwiek, Kamila Korzekwa, Aleksandra Tabiś, Jacek Bania, Gabriela Bugla-Płoskońska, Alina Wieliczko

**Affiliations:** 1 Department of Epizootiology with Clinic of Birds and Exotic Animals, Wroclaw University of Environmental and Life Sciences, 50-366 Wroclaw, Poland; katarzyna.molska@upwr.edu.pl; 2 Department of Microbiology, Institute of Genetics and Microbiology, Wroclaw University, S. 51-148 Wroclaw, Poland; kamila.korzekwa@uwr.edu.pl (K.K.); gabriela.bugla-ploskonska@uwr.edu.pl (G.B.-P.); 3 Department of Food Hygiene and Consumer Health Protection, Wroclaw University of Environmental and Life Sciences, 50-375 Wroclaw, Poland; aleksandra.tabis@upwr.edu.pl (A.T.); jacek.bania@upwr.edu.pl (J.B.)

**Keywords:** *Salmonella* Enteritidis, antimicrobial resistance, biofilm

## Abstract

*Salmonella enterica* ser. Enteritidis (*S. enterica* ser. Enteritidis) is the most frequently detected serovar in human salmonellosis, and its ability to produce a biofilm and the risk of transmission from animals and food of animal origin to humans are significant. The main aim of the present work was to compare *S. enterica* ser. Enteritidis strains isolated from poultry and human feces in terms of resistance profiles, prevalence of selected resistance genes, and their potential for biofilm formation, by assessing their biofilm growth intensity, the prevalence and expression of selected genes associated with this phenomenon, and the correlation between increased antimicrobial resistance and biofilm formation ability of the two tested groups of *S. enterica* ser. Enteritidis. This study showed a difference in antimicrobial resistance (minimal inhibitory concentration value) between *S. enterica* ser. Enteritidis groups; however, the majority of multidrug-resistant (MDR) strains were isolated from poultry (environmental samples from chicken broilers, turkey broilers, and laying hens). Differences in the prevalence of resistance genes were observed; the most common gene among poultry strains was *floR*, and that among strains from humans was *blaTEM*. *S. enterica* ser. Enteritidis strains isolated from poultry under the tested incubation conditions exhibited better biofilm growth than strains isolated from humans. A higher level of gene expression associated with the production of cellulose was only detected in the S48 strain isolated from poultry. On the other hand, increased expression of genes associated with quorum sensing was observed in two strains isolated from poultry farms and one strain isolated from human feces.

## 1. Introduction

According to the World Health Organization (WHO) [1], foodborne diseases remain a significant problem. Foodborne disease can be severe, especially among children, the elderly, and patients with immunosuppression. According to the latest European Food Safety Authority (EFSA) reports, *Salmonella* spp. continue to be the second most common cause of human infections and food poisoning associated with contaminated food [2]. *Salmonella* infections are a serious epidemiological and economic problem practically all over the world [3]. Human salmonellosis is an infectious disease that occurs in a variety of clinical forms and of varying severity (most often gastroenteritis), which are usually self-limiting. The etiological factor of salmonellosis is most often the two non-typhoid serovars: *Salmonella enterica* ser. Enteritidis (*S. enterica* ser. Enteritidis) and *Salmonella enterica* ser. Typhimurium (*S. enterica* ser. Typhimurium). Host–host transmission in most *Salmonella* serovars usually occurs via the fecal–oral route, and the most common source of infection with *Salmonella* spp. is animal products, in particular those related to poultry, including poultry meat, eggs, and confectionery containing eggs [4,5,6,7]. Salmonellosis is often asymptomatic in poultry, and carrier status may occur, which might present a zoonotic threat. In addition, infected individuals, including poultry, humans, cattle, and pigs, can become carriers and, as a result, excrete *Salmonella* spp. in their feces, functioning as a reservoir for this pathogen [8,9].

Another epidemiological problem is the growing frequency of drug-resistant strains, which can cause serious threats to public health. In recent years, more and more attention has been paid to the occurrence and threats resulting from the increasing resistance of Gram-negative bacteria isolated from poultry. These bacteria are resistant to quinolones, especially fluoroquinolones, which is the class of chemotherapeutics most commonly used in treating human salmonellosis infections, but they are also used in the fight against animal bacterial diseases due to their broad spectrum of antibacterial activity [10]. Another important problem is the increasing resistance to colistin, the last-line drug therapy for patients with multidrug-resistant (MDR) bacterial infections. According to the microbiological nomenclature, the MDR strain is defined as being non-susceptible (resistant or intermediate susceptible in vitro) to at least one antimicrobial from three or more groups of antibacterial drugs that are used to treat infections caused by the pathogen. Moreover, in developing countries, the increasing antibiotic resistance of *Salmonella* spp. strains is due to the irrational use of antimicrobials (abuse as well as misuse) in animal production [1]. Additionally, a link between the use of antimicrobial drugs in farm animals and the emergence of antimicrobial resistance among bacterial strains isolated from humans has been documented [11,12,13,14].

In addition to the global problem of increasing antimicrobial resistance described by the WHO, the ability of bacteria to form biofilms can cause therapeutic problems in human and veterinary medicine. It is worth noting that biofilms are becoming one of the important issues related to the food industry [15]. In relation to the genus *Salmonella*, important components of biofilms are cellulose [16] and curli fimbriae [17,18,19]. Cellulose is a key substance in the exopolysaccharide fraction in *Salmonella,* responsible for its sticky texture, and plays an important role in antimicrobial resistance of *Salmonella* biofilm. *Salmonella* can form biofilms on produced food; on poultry farm processing surfaces such as walls, floors, pipes, and drains; and on contact surfaces such as stainless steel, aluminum, nylon, rubber, plastic, polystyrene, or glass [20,21]. In addition, there is growing concern about the possibility of transmission of resistant bacterial strains, including those able to form biofilms, through the food production chain to consumers [22]. Considering the above-described issues related to increasing resistance, priority should be given to monitoring the resistance of bacterial pathogens that pose a threat to human health. Moreover, biofilm formation by *Salmonella* can hinder the eradication of microorganisms from the animal husbandry environment or production lines in the food industry and may pose a threat to consumer health.

Despite reports from Poland and across the world regarding strains of *S. enterica* ser. Enteritidis isolated from poultry or food of poultry origin [14,23,24,25,26,27], the comparative analysis of strains from the poultry farm environment with human feces samples from the aspects of antibiotic resistance and biofilm forming ability has not been evaluated so broadly before. Therefore, the main aim of the present work was to compare *S. enterica* ser. Enteritidis strains isolated from poultry and human feces in terms of resistance profiles, prevalence of selected resistance genes, and their potential for biofilm formation, by assessing their biofilm growth intensity, the prevalence and expression of selected genes associated with this phenomenon, and the correlation between increased antimicrobial resistance and biofilm formation ability of the two tested groups of *S. enterica* ser. Enteritidis.

## 2. Results

### 2.1. Molecular Identification of Salmonella enterica ser. Enteritidis

The presence of the *ompC* gene, characteristic of the *Salmonella* genus, and the *sdfI* gene, confirming that the bacteria belonged to the *S. enterica* ser. Enteritidis serovar, was detected in all 95 bacterial strains.

### 2.2. Antimicrobial Susceptibility and Resistance Gene Prevalence in Salmonella enterica ser. Enteritidis

The antimicrobial susceptibility results (MIC values) of all tested *S. enterica* ser. Enteritidis strains are presented in Table 1. All *S. enterica* ser. Enteritidis strains (100.0%) isolated from poultry were susceptible to gentamicin, ampicillin, tazobactam, cefotaxime, meropenem, azithromycin, tigecycline, and trimethoprim. In contrast, the vast majority of strains in this group were resistant to colistin (84.3%) and sulfamethoxazole (56.9%). In addition, 41.2% of poultry isolates were resistant to nalidixic acid and 21.6% to tetracycline. Moreover, a low percentage of *S. enterica* ser. Enteritidis strains isolated from poultry were resistant to ciprofloxacin and chloramphenicol—2.0 and 3.9%, respectively.

Among the *S. enterica* ser. Enteritidis strains isolated from humans, susceptibility to gentamicin, tazobactam, cefotaxime, meropenem, ciprofloxacin, azithromycin, tigecycline, and trimethoprim was similar to the group isolated from poultry—100.0% of strains were susceptible. In addition, all *S. enterica* ser. Enteritidis isolates from humans were susceptible to tetracycline, 95.5% to chloramphenicol, and 84.1% to sulfamethoxazole. The vast majority of strains in this group were resistant to colistin (79.5%) and nalidixic acid (56.8%).

**Figure 1:**
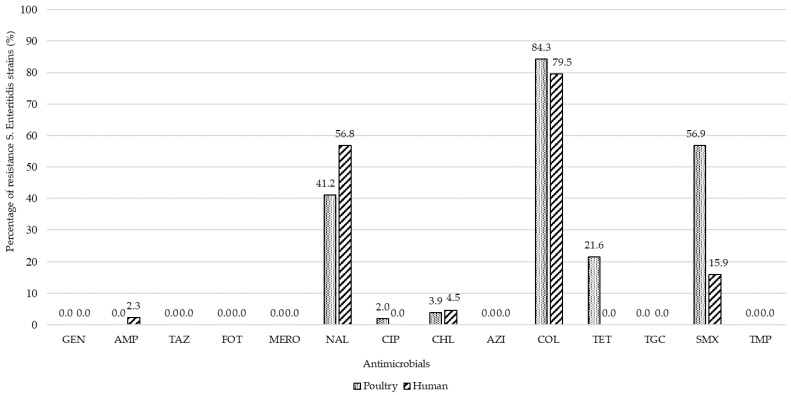
Percentage comparisons of *Salmonella enterica* ser. Enteritidis strains, isolated from poultry (n = 51) and humans (n = 44), resistant to selected antimicrobials. Explanation of abbreviations: GEN—gentamicin, AMP—ampicillin, TAZ—tazobactam, FOT—cefotaxime, MERO—meropenem, NAL—nalidixic acid, CIP—ciprofloxacin, CHL—chloramphenicol, AZI—azithromycin, COL—colistin, TET—tetracycline, TGC—tigecycline, SMX—sulfamethoxazole, and TMP—trimethoprim.

It should be emphasized that the highest percentage of *S. enterica* ser. Enteritidis strains, isolated from both poultry and humans, was resistant to colistin (84.3 and 79.5%, respectively) and to nalidixic acid (41.2 and 56.8%, respectively). In addition, only *S. enterica* ser. Enteritidis strains isolated from poultry were resistant to tetracyclines (21.6%), and the percentage of strains resistant to sulfamethoxazole was higher (56.9%) (Figure 1).

Based on the interpretation of The European Committee on Antimicrobial Susceptibility Testing (EUCAST) and Clinical and Laboratory Standards Institute (CLSI) breakpoint values, MICs were used to determine the eight multidrug-resistant (MDR) profiles of *S. enterica* ser. Enteritidis strains isolated from poultry and human feces. After analyzing the obtained results, a total of 18 strains with the MDR phenotype were isolated: 14 isolates from poultry, which constituted 27.5% of all collected strains of this group; and 4 strains derived from humans (9.1% strains of this group). The most common MDR profile was NAL COL SMX and included six strains, with four isolated from poultry and two from humans. The COL TET SMX profile was characteristic for four strains originating from the poultry farm environment, and the NAL COL TET SMX profile for three isolates also isolated from poultry. The NAL CHL COL TET SMX profile included only one strain from poultry (A09), but it is worth noting that resistance to most groups of antimicrobials was observed (Table 2).

**Figure 2:**
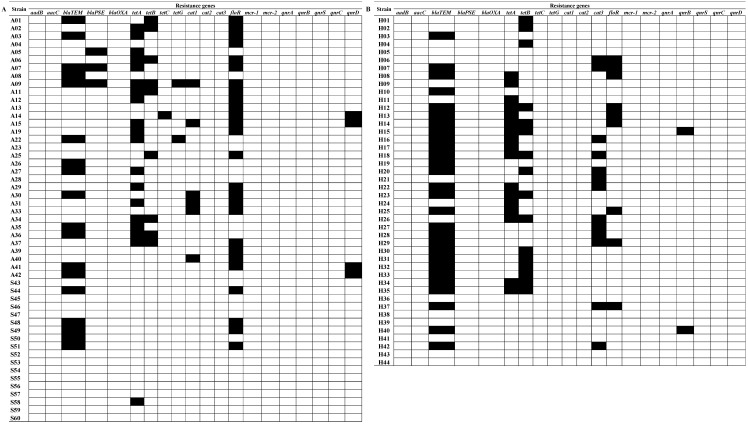
Prevalence of resistance genes to antimicrobials in *Salmonella enterica* ser. Enteritidis strains isolated from poultry sources (**A**) and human feces (**B**). White square: lack of resistance genes; black square: presence of resistance genes.

The most common resistance gene among strains from poultry was *floR* (51.0%), which conditions phenicol resistance (Figure 2). The prevalence of this gene among strains isolated from human feces was 20.5%. The most common resistance gene among strains isolated from human feces was *blaTEM* (63.6%), which confers beta-lactam resistance. The same gene was found in 35.3% of poultry strains. In addition, the *blaPSE* gene was present only in 5.9% of isolates from poultry. The prevalence of tetracycline resistance genes in both groups was as follows: the *tetA* gene was found in 37.3% of poultry strains and 38.6% of human strains, and *tetB* in 17.6 and 36.4% of strains, respectively. In contrast, *tetC* and *tetG* were detected only in isolates from poultry, and their prevalence was low—2.0 and 3.9%, respectively. Other resistance genes tested were *cat1*, found only in 11.8% of isolates from poultry, and *cat3*, found only in 29.5% of strains from humans. In both groups of *S. enterica* ser. Enteritidis strains, a very low prevalence of quinolone resistance genes was found, and no aminoglycoside or polymyxin resistance genes were found. It should be emphasized that 13.7% of *S. enterica* ser. Enteritidis strains isolated from poultry had resistance genes to three groups of tested antimicrobials, as much as 39.2% for two groups and 19.6% for one group. In contrast, 22.7, 36.4, and 25.0% of human isolate genes conferred resistance to three, two, and one group of antibiotics, respectively.

### 2.3. Salmonella enterica ser. Enteritidis Biofilm Formation

#### 2.3.1. Prevalence of Biofilm-Related Genes

In all tested *S. enterica* ser. Enteritidis strains from the poultry farm environment (n = 51) and human feces (n = 44), *csgD* and *sdiA* genes were detected. The *adrA* gene was found in 100.0% of poultry strains. Only in one *S. enterica* ser. Enteritidis human strain (H43) was the gene encoding the cellulose synthesis protein (*adrA*) not present.

#### 2.3.2. Assessment of *Salmonella enterica* ser. Enteritidis Morphotypes

**Figure 3:**
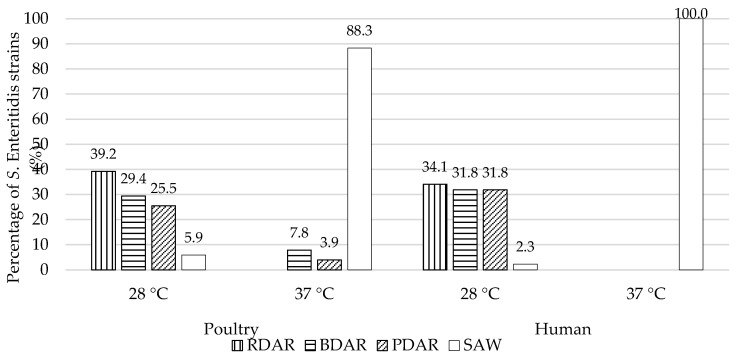
Percentage of *Salmonella enterica* ser. Enteritidis strains isolated from poultry and human feces by morphotype: RDAR (red, dry and rough), BDAR (brown, dry and rough), PDAR (pink dry and rough), and SAW (smooth and white) at 28 and 37 °C.

Among *S. enterica* ser. Enteritidis strains isolated from poultry, the occurrence of the RDAR morphotype at 28 °C was 39.2%. Under the same thermal conditions, the BDAR morphotype was noted in 15 (29.4%) strains. Slightly fewer, 25.5%, were cellulose-producing strains, with no synthesis of fimbriae curli components (PDAR). Only three strains (5.9%) showed the SAW morphotype at 28 °C, indicating a lack of synthesis of both important biofilm components. Prevalence of the RDAR, BDAR, and PDAR morphotypes among *S. enterica* ser. Enteritidis strains from human origin was assessed at very similar levels—34.1, 31.8, and 31.8%, respectively. Only one strain *S. enterica* ser. Enteritidis (H12) differed phenotypically in Congo Red Agar from the others, showing the SAW morphotype after incubation at 28 °C. After incubation at 37 °C, a different morphotype distribution was recorded among poultry and human *S. enterica* ser. Enteritidis strains. All human strains were included in the SAW morphotype (100.0%). Most isolates from the environment of poultry farms also had this morphotype (88.3%), and only 7.8 and 3.9% showed BDAR and PDAR morphotypes, respectively. In both groups, no RDAR morphotype was found at 37 °C, characteristic for the simultaneous production of cellulose and curli fimbriae (Figure 3).

#### 2.3.3. Biofilm Formation Assay

The results of the biofilm assay are presented in Table 3. The highest percentage of biofilm formation was in TBS medium at 28 °C (66.7% of poultry strains and 68.2% of strains isolated from humans, respectively). In turn, at 37 °C, most strains from both groups were characterized as weak biofilm formers, including 86.3% of isolates from poultry and 88.6% of isolates from humans. At 6 °C, it was observed that weak-producing EPS strains constituted the vast majority—78.4% and 65.9%, respectively. Most strains isolated from poultry (60.8%) at 28 °C were characterized by average biofilm growth intensity, while the highest percentage of isolates from humans (56.8%) formed biofilms poorly. At the temperature associated with the growth of human pathogenic bacteria (37 °C), only strains isolated from poultry showed medium (19.6%) or weak (80.4%) biofilm production. In contrast, 100.0% of strains isolated from humans were characterized by weak biofilm growth. At 6 °C, the vast majority of strains among both groups did not form biofilm or formed it weakly.

### 2.4. Expression of Genes Associated with Biofilm Production

**Figure 4:**
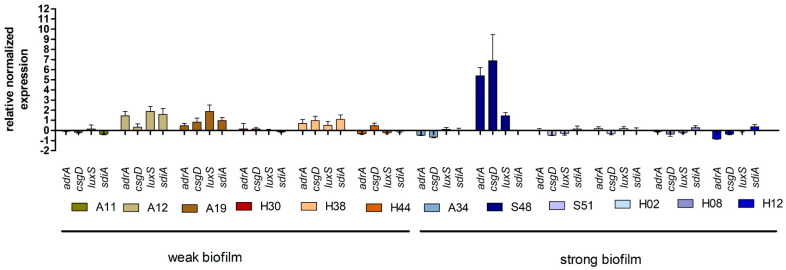
Relative expression of selected *adrA, csgD, luxA*, and *sdiA* genes, relative to the calibrator, during *Salmonella enterica* ser. Enteritidis biofilm culture at 28 °C.

The 12 *S. enterica* ser. Enteritidis strains selected for gene expression analysis were characterized by weak (n = 6) or strong (n = 6) biofilm growth in assay at 28 °C. All relative expression values ≤1.0 were interpreted as non-expression and >1.0 as increased expression. The high expression of genes responsible for regulating cellulose production in *Salmonella* spp., *adrA* and *csgD*, was observed only in strain S48 derived from poultry. A high expression level of *luxS*, involved in quorum sensing, was observed in A12 and A19 isolates from poultry. The expression of *sdiA,* a gene responsible for communication between bacterial cells, was high in A12 and H38 strains isolated from humans. It is worth noting that high expression levels of *adrA* and *csgD* were found in strain S48, showing a strong biofilm production capacity. In the case of *luxS* and *sdiA,* high expression levels were observed in strains H38, A12, and A19, which poorly formed a biofilm (Figure 4).

**Figure 5:**
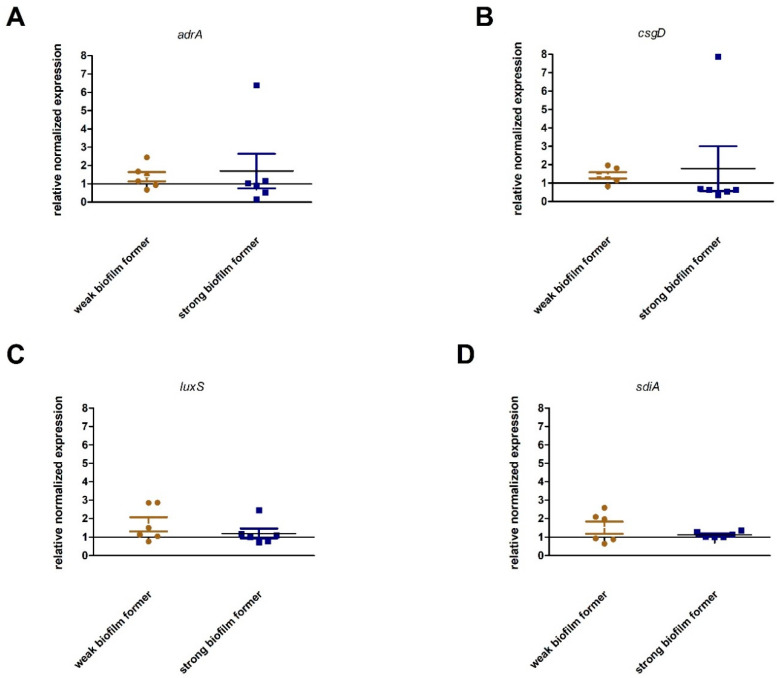
Relationship between the intensity of biofilm formation by *Salmonella enterica* ser. Enteritidis strains and the expression of biofilm-associated genes (**A**) adrA, (**B**) csgD, (**C**) luxS, and (**D**) sdiA. Mann–Whitney tests were used. No statistical difference was observed between groups.

None of the gene transcripts responsible for cellulose production and quorum sensing were significantly different between strong and weak biofilm producers (Figure 5).

**Figure 6:**
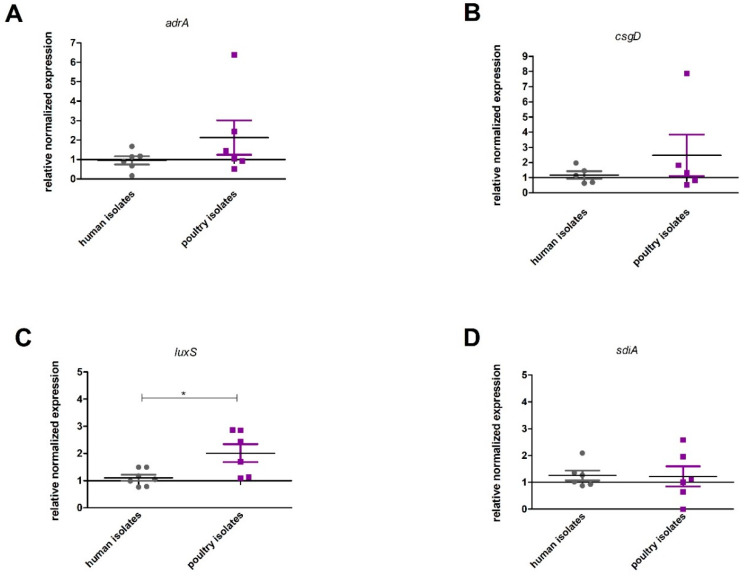
Relationship between the origin of the *Salmonella enterica* ser. Enteritidis strain and the expression of genes associated with biofilm formation (A adrA (*p* = 0.3939), B csgD (*p* = 0.8143) C luxS (*p* = 0,0381), and D sdiA, (*p* = 0.8182)). Quantification of results from three independent experiments; bars show means ± SD, Mann–Whitney tests; *p* ≤ 0.05 is shown by an asterisk *.

No significant difference in expression levels between the genes responsible for cellulose formation (*adrA p* = 0.3939, *csgD p* = 0.8410) and quorum sensing (*sdiA p* = 0.8182) was found. However, a significantly higher expression level (*p* = 0.0381) of the *luxS* gene was observed in strains isolated from poultry (Figure 6).

### 2.5. Statistical Analysis

Statistical analyses carried out in this study aimed to assess the occurrence of a statistically significant relationship between antibiotic resistance, morphotype, presence of resistance genes, and the ability to form biofilm by *S. enterica* ser. Enteritidis strains under the conditions selected for testing. The results showed there was no statistically significant relationship between the occurrence of the MDR phenotype and the ability to form biofilm under the tested conditions in either group of *S. enterica* ser. Enteritidis strains. A statistically significant relationship was observed only between the morphotypes (RDAR, PDAR, BDAR, and SAW) of *S. enterica* ser. Enteritidis strains isolated from humans and biofilm formation in TSB medium at 6 °C. For strains isolated from poultry farms, relationships between the type of morphotype and the culture in M9 medium at 28 and 37 °C as well as between the presence of resistance genes and the ability of biofilm formation by these strains in M9 medium at 6 °C were noted.

## 3. Discussion

In the European Union, salmonellosis is the second most common zoonotic disease, after campylobacteriosis, and the most commonly detected serovar among humans is *S. enterica* ser. Enteritidis. Antimicrobial resistance is currently a global problem [3], and, according to the WHO [1,28], it is extremely important to determine the prevalence of resistance genes in order to accurately describe the antibiotic resistance of microorganisms isolated from humans, animals, and food. Moreover, in the latest EFSA-ECDC antimicrobial resistance report [3], among *S. enterica* ser. Enteritidis strains recovered from humans in 2018, the most common were resistant to ampicillin, sulfonamides, tetracyclines, and ciprofloxacin. Therefore, one of the aims of our research was to determine and compare the antimicrobial resistance of *S. enterica* ser. Enteritidis strains from two sources. Moreover, the collected strains were analyzed for the discussed above antimicrobials.

Our results indicated that 11.4% of *S. enterica* ser. Enteritidis strains isolated from humans were susceptible to all tested antimicrobials, and in the case of poultry isolates only 2.0%. By contrast, Wasyl and Hoszowski [23] noted that 10.8% of *S. enterica* ser. Enteritidis strains isolated from the poultry farm environment, feed, and food were susceptible to antimicrobials.

Our research showed that all *S. enterica* ser. Enteritidis strains isolated from poultry and human feces were susceptible to gentamycin. Similar results were presented by Harb et al. [29], while other authors recorded strains being resistant to gentamycin from 7.1 to 12.5% [30,31,32]. In addition, the presence of *aadB* and *aacC* genes was not detected in our research, which was consistent with the results of other authors [33,34].

In our study, low resistance to beta-lactams was noted, which was previously demonstrated by Mąka et al. [24] among the isolates from food. On the other hand, Zhu et al. [32] noted resistance to ampicillin at 80% among *S. enterica* ser. Enteritidis strains from meat, but a low percentage of strains resistant to other beta-lactams. The high prevalence of the *blaTEM* gene (63.6% in our study) also corresponded with Zhu et al. [32]. It should be remembered that beta-lactam resistance may also be conditioned by other beta-lactamases in *Salmonella* spp. [35].

Tetracycline resistance, in our research, was demonstrated only in *S. enterica* ser. Enteritidis strains isolated from poultry (17.6%). In contrast, studies by Oliveira et al. [31] showed that approximately 12.5% of *S. enterica* ser. Enteritidis strains isolated from humans were resistant. *S. enterica* ser. Enteritidis resistance to tetracyclines was also found in studies by other authors, at rates of 1.0–100.0% [23,25,29]. It is worth pointing out that despite the lack of tetracycline-resistant strains among isolates from human feces, detection of resistance genes to this antimicrobial was noted among them. Comparable results were presented by Adesij et al. [36]. Similarly, the prevalence of beta-lactam resistance genes may have been related to the inactivity of the detected genes, but also to the potential for resistance under appropriate environmental conditions.

In the case of sulfamethoxazole, a large difference was observed in the percentage of resistant strains. These results were confirmed by Chen et al. [37] and Duffy et al. [38]. However, the prevalence rates presented by other authors [23,24,30,39] generally indicate a lower prevalence (0.0–10.0%) of sulfonamide resistance. It is worth noting that strains resistant to older generations of antimicrobials, such as tetracyclines and sulfamethoxazole, are most commonly isolated among *Salmonella* spp. [3,40].

Despite the high percentage of strains possessing phenolic resistance genes (poultry strains: *floR* 51.0% and *cat1* 11.8%; strains from humans: *cat3* 29.5% and *floR* 20.5%), no significant prevalence was observed among chloramphenicol-resistant strains. Similar results were presented by Zhu et al. [32]. The results herein and from other researchers may confirm the assumption that some genes are “turned off” in bacteria in vitro, but still have the potential to spread to other bacteria or to be involved in vivo, especially under the pressure of antibiotic selection [41].

It is worth noting that the majority of strains isolated from humans (56.8%) and poultry (41.2%) were resistant to nalidixic acid. Similar results (40.0%) were shown by Skarżyńska et al. [26]. Other authors noted a wide variety of nalidixic acid resistance (25.0–39.8%) in isolates from humans and food [25,29,31,42]. We noted that the prevalence of selected genes turned out to be very low, with a high percentage of nalidixic acid-resistant *Salmonella* strains. Other authors [10] did not record the presence of *qnrA* and *qnrC* genes among *S. enterica* ser. Enteritidis strains isolated from humans and from food samples—they found aac-(6’)-Ib. In summary, PMQR resistance, although more commonly reported in other serovars than *S. enterica* ser. Enteritidis [10,43], may have serious public health consequences, as fluoroquinolones are commonly used to treat adults with invasive diseases or other serious infections caused by bacteria of the genus *Salmonella*.

Colistin is not an antibiotic commonly used in medicine; nevertheless, its use in large-scale breeding of animals, including poultry, may lead to selection pressure, which may result in the creation of resistant strains. In this aspect, the results obtained in our research indicate a high prevalence and should be considered significant. The prevalence of colistin-resistant *S. enterica* ser. Enteritidis strains isolated from poultry (84.3%) and from humans (79.5%) was high compared to other national reports [14,25] and reports from other countries [30,44]. The cited authors did not note colistin-resistant strains in their studies. This major difference may be due to changes in CLSI and EUCAST recommendations [45,46] for colistin resistance reporting (currently MIC > 2.0 mg/µl). In this paper, *mcr-1* and *mcr-2* genes were not detected; however, colistin resistance genes (also *mcr3, mcr-4*, and *mcr5*) were previously identified in various *S*. *enterica* serovars, but not in *S. enterica* ser. Enteritidis [47,48,49,50].

It is worth considering that in human salmonellosis, ampicillin, fluoroquinolones, co-trimoxazole, third-generation cephalosporins and chloramphenicol are commonly used, while antibiotic therapy is prohibited and not implemented in the case of salmonellosis of poultry in the European Union. However, antimicrobials are used for other bacterial infections in poultry, and their presence in the environment may cause selective pressure for strains, including *Salmonella* spp. In recent years, among others in the United States, Europe and Asian countries, a decrease in ciprofloxacin susceptibility was observed among *Salmonella enterica* strains [3]. However, fluoroquinolones are still the most used chemotherapeutic agents in the treatment of salmonellosis in adults in Poland [51].

Currently, not only is resistance to individual antimicrobials monitored, but it is extremely important to determine the occurrence of multidrug-resistant bacterial strains. Literature analysis showed that the presence of multidrug-resistant *S. enterica* ser. Enteritidis strains is not as common compared to other *Salmonella* serovars [52]. In the results of our study (27.5% of MDR strains isolated from poultry and 9.1% of strains from humans), a lower percentage of MDR strains was noted compared to the report presented by Wasyl and Hoszowski [23] and Oliveira et al. [31]. However, the analysis of these results considered resistance to two or more groups of antimicrobials (not to three or more, as in the case of research in this study), which could generate such a high result. Comparable to the results of Oliveira et al. [31], the percentage of MDR strains (57.1%) was noted in the United States among isolates from imported food [53]. It is worth noting that in our own study, the A09 strain was described, isolated from poultry with an MDR phenotype, and was resistant to nalidixic acid, chloramphenicol, colistin, tetracycline, and sulfamethoxazole, which is a wide range of resistance (five groups of antimicrobials). Such a wide range of resistance in poultry isolates may increase the threat of *S. enterica* ser. Enteritidis zoonosis. In recent years, an increase in isolation of MDR *Salmonella* spp. (resistance to ampicillin, chloramphenicol, co-trimoxazole and tetracycline) the in European Union and other regions was observed [1,3]. For this purpose, the program “Communication from The Commission on a Community Strategy Against Antimicrobial Resistance” [54] implemented in European Union countries has aimed at controlling the use of antibiotics in animal husbandry as well as the rational use of antibiotics in treatment.

In our initial research assumptions, the tested *S. enterica* ser. Enteritidis strains might be similar in the field of antimicrobial resistance. The differences described above may result from un-matching sample collection dates. The poultry isolates were collected in the period 2009–2016 while the human feces isolates were collected in the period 2015–2017. In addition, poultry strains came from laboratories from two different regions of Poland. Moreover, by receiving strains isolated from human feces, it could not obtain information about the patient’s region (protection of personal data). These time–geographical discrepancies may have affected the differences in the results obtained.

*Salmonella enterica* as well as other species from the *Enterobacteriaceae* family are capable of adhering and forming biofilm on different kinds of materials during their life cycle [55]. Biofilms might be crucial to the survival of *Salmonella* in adverse environmental conditions, such as the poultry farm environment [56]. In the present research, the prevalence of *adrA* and *csgD* (cellulose production) as well as *adrA* and *sdiA* (quorum-sensing) genes was evaluated (100.0% of each gene). Only one strain isolated from human feces (H43) did not present *adrA*. The obtained results were consistent with Halatsi et al. [57] and Bhowmick et al. [58], who detected the genes described above not only in *S. enterica* ser. Enteritidis, but also in other *Salmonella* serovars. The presence of these genes and their expression might be useful in *Salmonella* biofilm production, via involvement in synthesis of EPS components and quorum-sensing communication.

The occurrence of morphotypes is dependent on the temperature and below 30 °C, production of crucial *Salmonella* biofilm elements is stimulated [16,19]. Solomon et al. [59] showed that 80% of *S. enterica* ser. Enteritidis isolates were characterized by the RDAR morphotype and the remainder by the SAW morphotype. On the other hand, the results by Lamas et al. [60] regarding *Salmonella* spp. from poultry farms showed a higher percentage of SAW morphotypes at 28 °C (77.1%) compared to our results, with a simultaneous 19.5% prevalence of the RDAR morphotype. Our results obtained at 37 °C (100.0% SAW morphotype among strains isolated from humans and 88.3% from poultry) were similar to those of Lamas et al. [60] and Römling et al. [19].

In compliance with data [61] describing that minimal media stimulates biofilm growth, TSB medium and M9 minimal medium were used for the present experiment. In the study by Lamas et al. [60], most strains from poultry farms (60.3%) were moderate biofilm formers at 28 °C in TSB, which was similar to the strains in our work (66.7%). On the other hand, Nair et al. [62] showed that *S. enterica* ser. Enteritidis (41.4%) isolated from poultry meat and human clinical samples produced a low level biofilm at 37 °C in TSB. Nonetheless, 14.3% of tested strains did not produce biofilm, which was not observed in our research. The obtained results of biofilm growth in M9 were significantly comparable to that of Lamas et al. [60], in which, at 28 °C, the majority (56.2%) of *S*. *enterica* strains from poultry showed moderate biofilm production. Moreover, incubation at 6 °C showed non-biofilm-forming strains (31.5%), as in our results, although 48.0% of tested strains by Lamas et al. [60] in these conditions showed weak biofilm formation. Research by Paz-Mendez et al. [63], contrary to our results, showed that all tested *S. enterica* strains produced biofilm in minimal media at 6 °C.

In the study of genes associated with biofilm formation expression, an increase was noted mainly in poultry strains. Gene expression was checked after 48 h incubation, which is in the earlier stages of biofilm formation; therefore, the relatively low gene expression associated with biofilm production may have been due to the initial phase of biofilm formation, when the ability to adhere to surfaces mainly is due to the cell surface characteristics, their hydrophobicity, and not to the EPS structure [64]. Transcription of biofilm-related genes is influenced by environmental factors such as acidic or low-nutrient conditions [65] as well as aerobic conditions during the incubation of *Salmonella* strains [66]. It is worth noting that among strains isolated from poultry (A12, A19, and S48), the increase in gene expression occurred in pairs: *adrA* + *csgD* and *sdiA* + *luxS*. Lamas et al. [67] showed a positive correlation in the expressions of *csgD*, *luxS,* and *sdiA* in anaerobic conditions. These results suggest a link between the expression of genes associated with biofilm detection and quorum sensing. Similarly, Wang et al. [21] achieved similar results in different culture media, suggesting a common expression of these genes. Confirming our results, Wang at al. [65] showed that in the early biofilm (72 h), investigated genes were downregulated. Upregulated expression of genes responsible for biofilm formation was assessed on the seventh day, i.e., in the mature biofilm [65]. Expression levels of *csgB* and *csgD* in TSB significantly increased after prolonged incubation, in line with the development of a mature biological layer [67], so it is worth expanding our own research in the future to analyze changes in expressions of selected genes after varying incubation times as well as in different media and temperature ranges. It is worth noting that many authors determine only the presence of a gene associated with biofilm formation, without checking the transcription level of individual genes [66]. The statistical analysis carried out showed no significant differences between genes responsible for either cellulose production or quorum sensing at the transcription level among the strains; therefore, it can be concluded that, among the randomly selected strains characterized by a strong biofilm growth, there was a high level of diversity relative to the genes tested.

## 4. Materials and Methods

### 4.1. Bacterial Strains

A total of 95 *Salmonella enterica* ser. Enteritidis strains were used in the present study and were isolated from two sources:

(1) Poultry *S. enterica* ser. Enteritidis strains (n = 51) isolated from the environment of chicken broilers, turkey broilers and laying hen farms. Isolates were obtained from two veterinary laboratories: AGRO-VET Laboratory in Wroclaw and SLW BIOLAB in Ostróda. These laboratories collected strains in the period 2009–2016. For our research, strains isolated from poultry are designated as “A” or “S”.
(2) Human *S. enterica* ser. Enteritidis strains (n = 44) isolated from human fecal samples were collected in the period 2015–2017 by Dialab Laboratory in Wroclaw. Strains isolated from humans are designated as “H”.

All collected strains were protected for further analysis and stored at −70 °C using Microbank^®^ (BioMaxima, Lublin, Poland).

### 4.2. Molecular Identification of Salmonella enterica ser. Enteritidis

All strains included in the present study were identified in accredited veterinary laboratories (AGRO-VET, Wroclaw and SLW BIOLAB, Ostróda) in accordance with the then applicable versions of the EN ISO 6579:2002 Microbiology of food and animal feeding stuffs—Horizontal method for the detection of *Salmonella* spp., EN ISO 6579:2002/Amd 1:2007 Microbiology of food and animal feeding stuffs—Horizontal method for the detection of *Salmonella* spp.—Amendment 1: Annex D: Detection of *Salmonella* spp. in animal feces and in environmental samples from the primary production stage and a medical diagnostic laboratory in accordance with its procedures. Molecular identification of *Salmonella enterica* ser. Enteritidis was carried out using PCR by amplifying the *ompC* sequence (F: 5′-ATCGCTGACTTATGCAATCG-3′; R:5′-CGGGTTGCGTTATAGGTCTG-3′), characteristic of the *Salmonella* genus, and the *sdfI* sequence (F:5′-TGTGTTTTATCTGATGCAAGAGG-3′; R: 5′-TGAACTACGTTCGTTCTTCTGG-3′), confirming the strain belonged to the *S. enterica* ser. Enteritidis serovar according to Freitas et al. 2010 [68].

### 4.3. Antimicrobial Susceptibility of Salmonella enterica ser. Enteritidis Strains

Antimicrobial susceptibility to selected antimicrobials (SMX—sulfamethoxazole, TMP—trimethoprim, NAL—nalidixic acid, CIP—ciprofloxacin, TET—tetracycline, MERO—meropenem, AZI—azithromycin, FOT—cefotaxime, CHL—chloramphenicol, TGC—tigecycline, TAZ—tazobactam, COL—colistin, AMP—ampicillin, and GEN—gentamicin) was tested with MIC Sensititre EU Surveillance *Salmonella*/*E. coli* EUVSEC commercial plates (Thermo Scientific, Waltham, MA, USA) according to the manufacturer’s instructions. *Escherichia coli* ATCC 25922 was used as the reference strain. Interpretation of results was performed in accordance with the recommendations of the European Committee on Antimicrobial Susceptibility Testing (EUCAST) version 9.0, 2019 [46], and Clinical Laboratory Standards Institute (CLSI) [45].

### 4.4. Antimicrobial Resistance Gene Detection

All *S. enterica* ser. Enteritidis strains were tested for 20 resistance-related genes, including those conferring resistance to aminoglicosides: *aadB* and *aacC*; beta-lactams: *blaTEM*, *blaPSE*, and *blaOXA*; tetracyclines: *tetA*, *tetB*, *tetC*, and tetG; phenicoles: *cat1*, *cat2*, *cat3*, and *floR*; polymyxin: *mcr*-1 and *mcr*-2; and quinolones: *qnrA*, *qnrB*, qnrC, *qnrD*, and *qnrS*. A list of primers used in research (Genomed, Poland) is presented in Table 1. For all simplex-PCR (*aadB*, *aacC*, *blaTEM*, *blaPSE*, *blaOXA*, *tetA*, *tetB*, *tetC*, *tetG*, *cat1*, *cat2*, *cat3*, *floR*, *qnrC*, and *qnrD*), duplex-PCR (*mcr-1* and *mcr-2*), and multiplex-PCR (*qnrA*, *qnrB*, and *qnrS*) reactions, the same cycling conditions were used with the following setup: 94 °C for 3 min and 30 cycles of denaturation (30 s, 94 °C), annealing (30 s, temperatures included in Table 4.), extension (30 s, 72 °C), and final extension (5 min, 72 °C). All PCRs were performed on a Thermal Cycler T100™ DNA Thermal Cycler (Bio-Rad, Irvine, CA, USA). The obtained products were subjected to electrophoretic separation in a 2.0% agarose gel and documented using a Gel-Doc UV Trans Illuminator System (Bio-Rad, Irvine, CA, USA). Quantity One software (Bio-Rad, Irvine, CA, USA) was used to analyze the electropherograms. Each PCR was carried out in at least two replicates. PCR products of the expected size were sequenced (Genomed, Warszawa, Poland) and analyzed using the BLAST database.

### 4.5. Salmonella enterica ser. Enteritidis Biofilm

#### 4.5.1. Prevalence of Biofilm-Related Genes

PCR detection of selected genes related to biofilm formation was carried out in accordance with Bhowmick et al. [58] and Halatsi et al. [57]. The *adrA* gene (F: 5′-ATGTTCCCAAAAATAATGAA-3′; R: 5′-TCATGCCGCCACTTCGGTGC-3′) encodes a protein that controls the level of cyclic di-GMP that regulates cellulose production. The *csgD* gene (F: 5′-TTACCGCCTGAGATTATCGT-3′; R: 5′-ATGTTTAATGAAGTCCATAG-3′) positively regulates *adrA* expression and is part of the csgDEFG operon, which increases the production of curli fimbriae proteins. The *sdiA* gene (F: 5′-AATATCGCTTCGTACCAC-3′; R: 5′-GTAGGTAAACGAGGAGCAG-3′) is involved in quorum sensing.

#### 4.5.2. *Salmonella enterica* ser. Enteritidis Morphotype on Congo Red Agar

The production of cellulose and curli fibers in *S. enterica* ser. Enteritidis was examined by streaking each strain on LB agar plates without salt, supplemented with 40 μg/mL Congo Red (Sigma-Aldrich, St. Louis, MO, USA) and 20 μg/mL brilliant blue (Sigma-Aldrich, St. Louis, MO, USA) and incubated for 48 h at 37 and 28 °C. The production of cellulose and curli fimbriae was determined based on colony morphology on Congo Red Agar: RDAR—red colony, expresses curli fimbriae and cellulose; BDAR—brown colony, expresses curli fimbriae; PDAR—pink colony, expresses cellulose; and SAW—no expression of curli fimbriae or cellulose morphotype [16,18,19]. *Escherichia coli* Nissle 1917 (EcN) strain, belonging to the collection of the Institute of Biotechnology, Brandenburg University of Technology in Germany, was used as a positive control.

#### 4.5.3. Biofilm Formation Assay

The dye crystal violet combines with the negatively charged components in biofilm extracellular polymeric substances (EPSs) and can, therefore, be used to determine the biofilm mass [61]. The experimental procedure, with modifications regarding the temperature and incubation time, was performed with the protocol developed by Lamas et al. [60]. Strains were precultured on LB (BIOCORP, Warszawa, Poland), and a single colony was used for inoculation. Incubation at 28 °C (stimulating cellulose and curli expression) and at 37 °C (optimal temperature for the growth of pathogenic bacteria) continued for 48 h and at 6 °C (using to store food in refrigerators) for 168 h (7 days). LB and M9 minimal media were used. Optical densities were measured at 590 nm (OD590) with a Spark microtiter reader (TECAN, Männedorf, Switzerland). Each strain was tested in quadruplicate in two independent experiments. Results were interpreted using classification by Stepanović et al. [61] as follows: OD ≤ ODc non-biofilm former; ODc < OD ≤ 2ODc weak biofilm former; 2ODc < OD ≤ 4ODc moderate biofilm former; 4ODc < OD strong biofilm former.

#### 4.5.4. Expression of Genes Associated with Biofilm Production

In order to compare the expression levels of *csgD*, *adrA*, *sdiA,* and *luxS* (the primers listed in Table 5), genes in 12 strains, which included weak biofilm formers ((n = 6) H30, H38, H44, A11, A12, and A19) and strong biofilm formers ((n = 6) H02, H08, H12, A34, S48, and S51) at 28 °C were used. Both groups included representative human and poultry strains (six human isolates and an equal number of isolates from poultry). Each strain was cultured in duplicate in a volume of 40 mL TSB medium (condition of bacterial culture—the density of the culture and general condition corresponded to the biofilm formation assay) for 48 h at 28 °C in Greiner Cellstar Dish plates (100 × 20 mm) (MERCK, Warszawa, Poland), which are made of the same type of plastic as a plates used in the biofilm formation assay. After incubation, the plates were washed twice, and the adhering bacterial cells were recovered by scraping and suspension in 1 mL of sterile PBS (Fisher BioReagents™, Waltham, MA, USA). After centrifugation (2000 g, 5 min), pellets were used for RNA isolation. RNA was extracted using RNA Mini Plus (A&A Biotechnology, Poland) following the manufacturer’s recommendations. The RNA concentration and purity (A260 nm/A280 nm ratios) were obtained spectrophotometrically. (The integrity after isolation was checked by 1.0% agarose gel electrophoresis.) Contaminated DNA was removed using DNase I (Sigma-Aldrich, St. Louis, MO, USA) in a volume of 50 µL at 37 °C for 2 h. Total cDNA was amplified from 1 µg of RNA with SuperScript III (Invitrogen, Waltham, MA, USA) according to the manufacturer’s instructions. Synthesis was performed with Random Hexamers in the following parameters: 5 min at 25 °C, 50 min at 50 °C, and 15 min at 70 °C. Real-time PCR reactions were performed in a thermocycler CFX-Connect™ Real-Time System (Bio-Rad, Irvine, CA, USA) according to the protocol developed by Lamas et al. [66]. After optimization, the reaction efficiency was higher than 97% for every tested gene. The reaction mixture contained 1 μL of template cDNA, 0.5 μM of each primer (listed in Table 5.), 10 μL of SsoFast EvaGreen Supermix, and water up to 20 μL, and the protocol was as follows: 95 °C for 30 s, followed by 35 cycles of 95 °C for 10 s and 60 °C for 15 s. Specificity of PCR was evaluated by melt-curve analysis in a temperature range 95–58 °C, and analysis of PCR products on agarose gel was performed for each reaction. The mRNA levels of all target genes were normalized to the 16S rRNA gene as the most stable in biofilm production in *Salmonella* [66]. Relative transcript levels were calculated according to Pfaffl [70] using Bio-Rad CFX Manager 3.0, and statistical calculations were made using Statistica 12.5 software (Stat Soft Polska Sp. z.o.o.). The non-parametric Mann–Whitney test was used for comparisons between biofilm-forming groups.

### 4.6. Statistical Analysis

The analysis was performed at a significance level of 5% using PQStat ver. 1.6.2 for Windows (PQStat Software © 2016), using Fisher’s exact test, and TIBCO Software Inc. (2017), Statistica (data analysis software system), version 13.3, for Spearman rank correlation.

## 5. Conclusions

*S. enterica* ser. Enteritidis strains isolated from poultry and humans were similar in terms of the high prevalence of resistance to nalidixic acid. Similarly, a high colistin resistance was described, while no *mcr-1* and *mcr-2* genes could be detected. However, a high percentage of sulfamethoxazole-resistant strains was noted among isolates from poultry, and tetracycline resistance was described only within this group. We noted the presence of MDR strains, but it is worth emphasizing that most MDR phenotypes were described among *S. enterica* ser. Enteritidis strains isolated from poultry. The most common MDR profiles in this group were COL NAL SMX and COL TET SMX. In our study, the differences in the prevalence of resistance genes to beta-lactam, tetracycline, and phenicol between isolates from two sources were described; the most frequent gene among strains isolated from poultry was *floR*, and among strains from humans, *blaTEM*. To summarize biofilm production research, it should be noted that *S. enterica* ser. Enteritidis strains isolated from poultry under the tested conditions in vitro were characterized phenotypically as better biofilm formers.

This study did not show statistically significant differences in the transcriptomic levels between the two *S. enterica* ser. Enteritidis groups in terms of biofilm formation and the expression of genes responsible for cellulose production and quorum sensing. A similar observation on the expression of genes responsible for cellulose production as well as quorum-sensing genes in poultry and human strains was made. Moreover, a statistically significant correlation was not found between the MDR phenotype and the ability to form biofilm in the tested in vitro conditions in both analyzed *S. enterica* ser. Enteritidis strains.

## Figures and Tables

**Table 1:** Antimicrobial dilution range for 95 *Salmonella enterica* ser. Enteritidis strains isolated from poultry sources and human feces according to minimal inhibitory concentration (MIC) values.

Antimicrobial Agent	Antimicrobial Dilution Range (µg/mL)																
	0.015	0.03	0.06	0.12	0.25	0.5	1	2	4	8	16	32	64	128	256	512	1024
**Poultry (n = 51)**																	
Gentamicin	x	x	x	x	x	45	3	1	2				x	x	x	x	x
Ampicillin	x	x	x	x	x	x	11	35	4	1				x	x	x	x
Tazobactam	x	x	x	x	x	49	2				x	x	x	x	x	x	x
Cefotaxime	x	x	x	x	49		2			x	x	x	x	x	x	x	x
Meropenem	x	51										x	x	x	x	x	x
Nalidixic acid	x	x	x	x	x	x	x	x	24	1	5	1	4	(16 *)	x	x	x
Ciprofloxacin	2	18	1	2	12	15	1				x	x	x	x	x	x	x
Chloramphenicol	x	x	x	x	x	x	x	x	x	49	1		1		x	x	x
Azithromycin	x	x	x	x	x	x	x		16	35				x	x	x	x
Colistin	x	x	x	x	x	x	6	2	29	14		x	x	x	x	x	x
Tetracycline	x	x	x	x	x	x	x	34	4	2	4	5	1(1 *)	x	x	x	x
Tigecycline	x	x	x	x		36	15				x	x	x	x	x	x	x
Sulfamethoxazole	x	x	x	x	x	x	x	x	x			11	8	2	1	1	(28 *)
Trimethoprim	x	x	x	x	12	36	12	1					x	x	x	x	x
**Human (n = 44)**																	
Gentamicin	x	x	x	x	x	39	5						x	x	x	x	x
Ampicillin	x	x	x	x	x	x	5	35	3			1		x	x	x	x
Tazobactam	x	x	x	x	x	44					x	x	x	x	x	x	x
Cefotaxime	x	x	x	x	44					x	x	x	x	x	x	x	x
Meropenem	x	44										x	x	x	x	x	x
Nalidixic acid	x	x	x	x	x	x	x	x	18	1		2	15	(8 *)	x	x	x
Ciprofloxacin	1	18	2	1	13	9					x	x	x	x	x	x	x
Chloramphenicol	x	x	x	x	x	x	x	x	x	42	2				x	x	x
Azithromycin	x	x	x	x	x	x	x		7	37				x	x	x	x
Colistin	x	x	x	x	x	x		9	13	22		x	x	x	x	x	x
Tetracycline	x	x	x	x	x	x	x	37	6	1				x	x	x	x
Tigecycline	x	x	x	x		27	17				x	x	x	x	x	x	x
Sulfamethoxazole	x	x	x	x	x	x	x	x	x			14	22	1			(7 *)
Trimethoprim	x	x	x	x	12	30	2						x	x	x	x	x

Breakpoints were adopted from The European Committee on Antimicrobial Susceptibility Testing (EUCAST) and Clinical and Laboratory Standards Institute (CLSI), when available; breakpoints for azithromycin and tigecycline are unavailable; numbers: number of strains with each MIC value; lack of number; strains with each MIC value are not described; grey square: resistant to an antimicrobial; white square: susceptible to an antimicrobial; x: the dilution range marked in this MIC Sensititre EU Surveillance *Salmonella*/*E. coli* EUVSEC commercial plates was not examined for an antimicrobial; * MIC value greater than maximum tested.

**Table 2:** MDR profiles of *Salmonella enterica* ser. Enteritidis (n = 18) phenotypes.

No.	Multidrug Resistance Profile	*S. enterica* ser. Enteritidis Strains
1	NAL CHL COL TET SMX	A09
2	NAL CIP COL SMX	A37
3	NAL CHL COL SMX	A40
4	NAL COL TET SMX	S47, S55, S56
5	NAL COL SMX	A12, A41, A42, S60, H24, H34
6	COL TET SMX	A22, A23, A25, A31
7	CHL COL SMX	H33
8	AMP NAL COL	H10

A or S: strain isolated from poultry; H: strain isolated from human feces.

**Table 3:** The number and percentage of *Salmonella enterica* ser. Enteritidis strains isolated from poultry (n = 51) and humans (n = 44) and biofilm growth intensity in TSB and M9 media at 28, 37, and 6 °C.

**Intensity at 28 °C**	**TSB**				**M9**			
	**Poultry**		**Human**		**Poultry**		**Human**	
	**n**	**%**	**n**	**%**	**n**	**%**	**n**	**%**
0	0	0.0	0	0.0	0	0.0	0	0.0
1	4	7.8	6	13.6	12	23.5	25	56.8
2	34	66.7	30	68.2	31	60.8	18	40.9
3	13	25.5	8	18.2	8	15.7	1	2.3
**Intensity at 37 °C**	**TSB**				**M9**			
	**Poultry**		**Human**		**Poultry**		**Human**	
	**n**	**%**	**n**	**%**	**n**	**%**	**n**	**%**
0	0	0.0	0	0.0	0	0.0	0	0.0
1	44	86.3	39	88.6	41	80.4	44	100.0
2	7	13.7	5	11.4	10	19.6	0	0.0
3	0	0.0	0	0.0	0	0.0	0	0.0
**Intensity at 6 °C**	**TSB**				**M9**			
	**Poultry**		**Human**		**Poultry**		**Human**	
	**n**	**%**	**n**	**%**	**n**	**%**	**n**	**%**
0	9	17.7	11	25.0	20	39.2	17	38.6
1	40	78.4	29	65.9	31	60.8	26	59.1
2	2	3.9	4	9.1	0	0.0	1	2.7
3	0	0.0	0	0.0	0	0.0	0	0.0

0: non-biofilm former; 1: weak biofilm former; 2: moderate biofilm former; 3: strong biofilm former.

**Table 4:** Primers used in PCR to detect resistance genes in *Salmonella enterica* ser. Enteritidis strains.

Gene	Sequence	PCR Product Size (bp)	Annealing Temperature (°C)	References
*aadB*	F: 5′-GAGCGAAATCTGCCGCTCTGG-3′ R: 5′-CTGTTACAACGGACTGGCCGC-3′	319	61	[34]
*aacC*	F: 5′-GGCGCGATCAACGAATTTATCCGA-3′ R: 5′-CCATTCGATGCCGAAGGAAACGAT-3′	488	58	
*blaTEM*	F: 5′-CATTTCCGTGTCGCCCTTAT-3′ R: 5′-TCCATAGTTGCCTGACTCCC-3′	793	55	
*blaPSE*	F: 5′-AATGGCAATCAGCGCTTCCC-3′ R: 5′-GGGGCTTGATGCTCACTACA-3′	586	59	
*blaOXA*	F: 5′-ACCAGATTCAACTTTCAA-3′ R: 5′-TCTTGGCTTTTATGCTTG-3′	590	55	
*tetA*	F: 5′-GCTACATCCTGCTTGCCTTC-3′ R: 5′-CATAGATCGCCGTGAAGAGG-3′	210	55	
*tetB*	F: 5′-TTGGTTAGGGGCAAGTTTTG-3′ R: 5′-GTAATGGGCCAATAACACCG-3′	659	53	
*tetC*	F: 5′-CTTGAGAGCCTTCAACCCAG-3′ R: 5′-ATGGTCGTCATCTACCTGCC-3′	417	56	
*tetG*	F: 5′-GCTCGGTGGTATCTCTGCTC-3′ R: 5′-AGCAACAGAATCGGGAACAC-3′	468	58	
*cat1*	F: 5′-CCTATAACCAGACCGTTCAG-3′ R: 5′-TCACAGACGGCATGATGAAC-3′	491	56	
*cat2*	F: 5′-CCGGATTGACCTGAATACCT-3′ R: 5′-TCACATACTGCATGATGAAC-3′	456	56	
*cat3*	F: 5′-CCCACAATTCACCGTATTCC-3′ R: 5′-GAACCTGTACTGAGAGCGGC-3′	310	58	
*floR*	F: 5′-AACCCGCCCTCTGGATCAAGTCAA-3′ R: 5′-CAAATCACGGGCCACGCTGTATC-3′	548	60	
*mcr-1*	F: 5′-CGGTCAGTCCGTTTGTTC-3′ R: 5′-CTTGGTCGGTCTGTAGGG-3′	309	59	[69]
*mcr-2*	F: 5′-TGTTGCTTGTGCCGATTGGA-3′ R: 5′-AGATGGTATTGTTGGTTGCTG-3′	567	59	
*qnrA*	F: 5′-ATTTCTCACGCCAGGATTTG-3′ R: 5′-GATCGGCAAAGGTTAGGTCA-3′	516	56	[10]
*qnrB*	F: 5′-GATCGTGAAAGCCAGAAAGG-3′ R: 5′-ACGATGCCTGGTAGTTGTCC-3′	469	56	
*qnrC*	F: 5′-GGGTTGTACATTTATTGAATCG-3′ R: 5′-CACCTACCCATTTATTTTCA-3′	307	54	
*qnrD*	F: 5′-CGAGATCAATTTACGGGGAATA-3′ R: 5′-AACAAGCTGAAGCGCCTG-3′	582	52	
*qnrS*	F: 5′-ACGACATTCGTCAACTGCAA-3′ R: 5′-TAAATTGGCACCCTGTAGGC-3′	417	56	

**Table 5:** Primers used to assess the associated genes with biofilm expression in *Salmonella enterica*.

Gene/Region	Sequence
*csgD*	F: 5′-TCCTGGTCTTCAGTAGCGTAA-3′ R: 5′-TATGATGGA AGCGGATAAGAA-3′
*adrA*	F: 5′-GAAGCTCGTCGCTGGAAGTC-3′ R: 5′-TTCCGCTTAATTTAATGGCCG-3′
*luxS*	F: 5′-ATGCCATTATTAGATAGCTT-3′ R: 5′-GAGATGGTCGCGCATAAAGCCAGC-3′
*sdiA*	F: 5′-AATATCGCTTCGTACCAC-3′ R: 5′-GTAGGTAAACGAGGAGCAG-3′
16S rRNA reference gene	F: 5′-AGGCCTTCGGGTTGTAAAGT-3′ R: 5′-GTTAGCCGGTGCTTCTTCTG-3′
